# Beaver: Nature's ecosystem engineers

**DOI:** 10.1002/wat2.1494

**Published:** 2020-11-27

**Authors:** Richard E. Brazier, Alan Puttock, Hugh A. Graham, Roger E. Auster, Kye H. Davies, Chryssa M. L. Brown

**Affiliations:** ^1^ Department of Geography University of Exeter Devon UK

**Keywords:** beaver, catchment management, ecological restoration, ecosystem engineers, hydrology

## Abstract

Beavers have the ability to modify ecosystems profoundly to meet their ecological needs, with significant associated hydrological, geomorphological, ecological, and societal impacts. To bring together understanding of the role that beavers may play in the management of water resources, freshwater, and terrestrial ecosystems, this article reviews the state‐of‐the‐art scientific understanding of the beaver as the quintessential ecosystem engineer. This review has a European focus but examines key research considering both *Castor fiber*—the Eurasian beaver and *Castor canadensis*—its North American counterpart. In recent decades species reintroductions across Europe, concurrent with natural expansion of refugia populations has led to the return of *C. fiber* to much of its European range with recent reviews estimating that the *C. fiber* population in Europe numbers over 1.5 million individuals. As such, there is an increasing need for understanding of the impacts of beaver in intensively populated and managed, contemporary European landscapes. This review summarizes how beaver impact: (a) ecosystem structure and geomorphology, (b) hydrology and water resources, (c) water quality, (d) freshwater ecology, and (e) humans and society. It concludes by examining future considerations that may need to be resolved as beavers further expand in the northern hemisphere with an emphasis upon the ecosystem services that they can provide and the associated management that will be necessary to maximize the benefits and minimize conflicts.

This article is categorized under:

- Water and Life > Nature of Freshwater Ecosystems

## Graphical abstract

This article reviews the state‐of‐the‐art scientific understanding of the beaver as an ecosystem engineer. It summarizes how beaver impact: (a) ecosystem structure and geomorphology, (b) hydrology and water resources, (c) water quality, (d) freshwater ecology, and (e) humans and society.

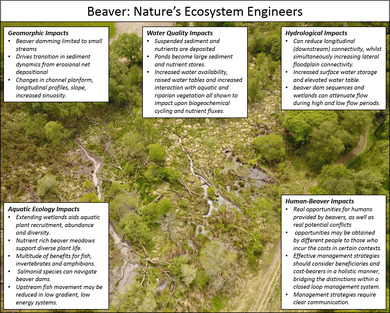

## 1 INTRODUCTION

Over millions of years, beavers (*Castoridae)* have developed the ability to modify ecosystems profoundly to meet their ecological needs. In doing so, they also provide valuable habitats for many other species that thrive in wetlands. They engineer ecosystems by building dams, which retain ponds, full of sediment, nutrients, plants, and wildlife. These dams slow the flow of water, reducing peak flows downstream (Puttock, Graham, Cunliffe, Elliott, & Brazier, 2017), storing and gently releasing water in times of drought (Hood & Bayley, 2008). Beavers excavate canals, laterally across floodplains, to access and transport food and building resources, enhancing floodplain connectivity, and geomorphic dynamism (Gorczyca, Krzemień, Sobucki, & Jarzyna, 2018; Pollock et al., 2014). They coppice trees, providing deadwood habitat and allowing sunlight to reach understory vegetation which in turn responds in abundance and diversity (Law, Gaywood, Jones, Ramsay, & Willby, 2017), providing rich habitat for insects, birds, bats, and amphibians (Dalbeck, Hachtel, & Campbell‐Palmer, 2020; Stringer & Gaywood, 2016; Willby, Law, Levanoni, Foster, & Ecke, 2018). Beavers were once present throughout Europe, Asia, and North America in large numbers, managing water resources, working with natural processes, supporting the healthy functioning of freshwaters—the very definition of a keystone species.

Consider the potential implications of removing such an animal from our ecosystems. Large areas of stored surface water are lost, rivers flow faster, becoming flashy in times of flood and with lower baseflows in times of drought. Woody debris, carbon in water—an essential building block of life in ponds, streams, rivers, estuaries, and marine environments is reduced, undermining the food‐chains that it supported. Wetlands dry up, wildlife move on, or are possibly lost from ecosystems entirely. During the Anthropocene, our catchments have largely become a product of human activity that realizes all of these implications, with associated additional pressures including; hydrological extremes, diffuse pollution, and soil erosion (Hewett, Wilkinson, Jonczyk, & Quinn, 2020). The natural disturbance and dynamic equilibrium maintained by beaver activity drives geomorphic and ecological complexity, in their absence, riparian ecosystems have taken on a simpler form both in terms of their structure and their function (Brown et al., 2018).

In the Northern hemisphere, beavers were hunted to near extinction and extirpated entirely in countries such as Great Britain (GB) about 400 years ago (Conroy & Kitchener, 1996). Thus, our living memory of what beaver‐lands were like, is limited, in landscapes where natural recolonizations or reintroductions are now taking place. Our understanding of how *other* species co‐existed with beavers, many of them dependent upon wetlands such as beaver ponds, is similarly limited. There is thus a requirement to understand the impact of beavers in contemporary ecosystems, particularly in landscapes that, since their extirpation, have been over‐exploited, degraded, and altered by intensive farming and urban development.

To bring together understanding of the role that beavers may play in the management of water resources, freshwater, and terrestrial ecosystems, this paper reviews the state‐of‐the‐art scientific understanding of the beaver as the quintessential ecosystem engineer. We focus upon research considering both *Castor fiber*—the Eurasian beaver and *Castor canadensis*—its North American counterpart, as they re‐establish in ecosystems within which their numbers were decimated and are reintroduced or return to ecosystems from where they were extirpated, due to their high‐value fur (for hats), castoreum (as a painkiller and perfume)—Nolet and Rosell (1998), and their scaly tail, which led the Catholic church to classify beavers as a fish—fit for consumption on Fridays and Saints days (Coles, 2006; Kitchener & Conroy, 1997; Manning et al., 2014).

The remaining two species of beaver are related to pre‐historic *Castoridae* which included as many as 40 species, for example, the giant beaver (*C. Castorides spp*; Martin, 1969) and the terrestrial *C. Paleocastor spp*, famed for its spiralized burrows (Martin & Bennett, 1977). Today, the two extant species of beaver are genetically distinct with differing numbers of chromosomes (Kuehn, Schwab, Schroeder, & Rottmann, 2000). Despite their genetic and minor physiological differences, there are many similarities between the species. For example, they are visually similar and difficult to differentiate by sight alone (Kuehn et al., 2000). Until relatively recently, it was considered that the North American beaver had a tendency to build dams and lodges more frequently and of a greater size than the Eurasian beaver, but it has now been shown by Danilov and Fyodorov (2015) that, under the same environmental conditions, the building behavior of the two species does not differ.

In recent decades species reintroductions across Europe, followed by natural expansion has led to the return of *C. fiber* to much of its Eurasian range (Halley, Rosell, & Saveljev, 2012) with a recent review of national population studies, estimating that the *C. fiber* population in Europe numbers over 1.5 million individuals (Halley et al., 2012). As such, there is an increasing need for understanding of the impacts of beaver in intensively populated and managed modern European landscapes. This review focuses on Europe and *C. fiber* but draws on relevant research into *C. canadensis* in North America. The review summarizes how beaver impact: (a) ecosystem structure and geomorphology, (b) hydrology and water resources, (c) water quality, (d) freshwater ecology, and (e) humans and society. It concludes by examining future scenarios that may need to be considered as beavers expand in the northern hemisphere with an emphasis upon the ecosystem services that they can provide and the associated management that will be necessary to maximize the benefits and minimize conflicts.

## 2 BEAVER IMPACT UPON THE ENVIRONMENT—CONTEMPORARY UNDERSTANDING

### 2.1 Impacts of beaver upon geomorphology

#### 2.1.1 Overview

We take this opportunity to revisit Gurnell's (1998) review on the hydrogeomorphological effects of beaver, which provides an excellent foundation for our understanding. Beavers, as ecosystem engineers, have a marked influence upon the terrestrial and riverine environments that they occupy (Westbrook, Cooper, & Baker, 2011). Beavers are primary agents of zoogeomorphic processes; here we acknowledge their influence upon river form and process (Johnson et al., 2020) and discuss recent literature on the impacts of beaver on hydrogeomorphology.

#### 2.1.2 Canal and burrow excavation

Beavers are well known for their construction of impressive lodges, sometimes as tall as 3 m (Danilov & Fyodorov, 2015), but beavers, especially in river systems, typically excavate bank burrows in which to establish dwellings (Collen & Gibson, 2000; Rosell, Bozer, Collen, & Parker, 2005). Beavers often excavate multiple burrows in a single territory, which can contribute significant volumes of sediment to a watercourse (de Visscher, Nyssen, Pontzeele, Billi, & Frankl, 2014; Lamsodis & Ulevičius, 2012) and also create areas of weakness which can lead to localized erosion and, in some instances, the collapse of earthen flood embankments (Harvey, Henshaw, Brasington, & England, 2019).

Beavers commonly dig shallow channels, often referred to as canals, which extend laterally from beaver ponds. These structures enable beavers to access food and building resources more easily (Butler, 1991; Gurnell, 1998). Often developing into dense networks, these canals contribute significantly to the local hydrogeomorphology of floodplains, creating hydraulic roughness, tortuous flow paths, and complex topography in otherwise planar landscapes (Hood & Larson, 2015). Like burrows, these canals may act as a source of fine sediment (Lamsodis & Ulevičius, 2012; Puttock, Graham, Carless, & Brazier, 2018) or, in the event of significant overbank flows and floodplain inundation, sites of deposition. It is interesting to consider that early humans might have moved over (crossing channels on beaver dams) and through beaver landscapes crisscrossed by canals, observing beaver transporting woody building materials by water with ease, and subsequently learning to do so themselves (Coles, 2006).

#### 2.1.3 Woody debris contribution

Woody debris is a key driver of geomorphic complexity, has been shown to be a fundamental aspect of “natural” stream geomorphology and a critical habitat for aquatic life (Collen & Gibson, 2000; Gurnell, Piégay, Swanson, & Gregory, 2002; Harvey, Henshaw, Parker, & Sayer, 2018; Thompson et al., 2018; Wohl, 2014, 2015). Beaver increase the rate of both large and small woody material contribution to river systems (Gurnell et al., 2002). In small streams, the large woody material (for example felled trees) is less mobile and often remains in place, exerting a strong influence on geomorphic processes, increasing bed heterogeneity through promoting localized scour and deposition (Gurnell et al., 2002). The contribution of smaller woody fragments or cuttings has been shown to significantly increase willow (*Salix spp*) recruitment due to the provision of propagules, which can establish on gravel/sand bars (Levine & Meyer, 2019). This increases the stability of depositional features and promotes rates of aggradation and bed/bank stability.

#### 2.1.4 Dam building

Beavers have a preference for habitats with deep, slow‐flowing water, to feel safe from predators (Collen & Gibson, 2000; Hartman & Tornlov, 2006; Swinnen, Rutten, Nyssen, & Leirs, 2019). Therefore, their dam‐building activity is typically restricted to lower‐order streams where stream power is limited (Graham et al., 2020; Gurnell, 1998; Macfarlane et al., 2015; Rosell et al., 2005) and water depths may not be sufficient (normally <0.7 m depth) for beaver movement and security. When dam building does occur, it increases the area of lentic (still freshwater) habitats in systems that are typically dominated by lotic (free‐flowing freshwater) habitats (Hering, Gerhard, Kiel, Ehlert, & Pottgiesser, 2001). Damming typically reduces downstream connectivity, and conversely increase lateral connectivity, forcing water sideways into neighboring riparian land, inundating floodplains, and creating diverse wetland environments (Hood & Larson, 2015) as well as contributing to soil and groundwater recharge (Westbrook, Cooper, & Baker, 2006). Dams vary significantly in their size and structure depending on physical factors such as hydrology, topography, and building materials but also ecological factors (Graham et al., 2020). Hafen, Wheaton, Roper, Bailey, and Bouwes (2020) found that primary dams, that maintained a lodge pond, were significantly larger than secondary dams, which are used to improve mobility and the transport of woody material, concluding that beaver ecology, in addition to channel characteristics, exerts a primary control on dam size.

#### 2.1.5 Agents of erosion

Erosion often occurs at the base of dams, due to a localized increase in gradient and stream power (Gurnell, 1998; Lamsodis & Ulevičius, 2012). Woo and Waddington (1990) observed that flow across the dam crest may be concentrated in gaps, enhancing erosion of the stream bed and banks downstream of the dam, forming plunge pools, and widening the channel, respectively. Lamsodis and Ulevičius (2012) observed the geomorphic impacts of 242 dams in lowland agricultural streams in Lithuania; of which, 13 (5.4%) experienced scour around the periphery of the dam.

**FIGURE 1:**
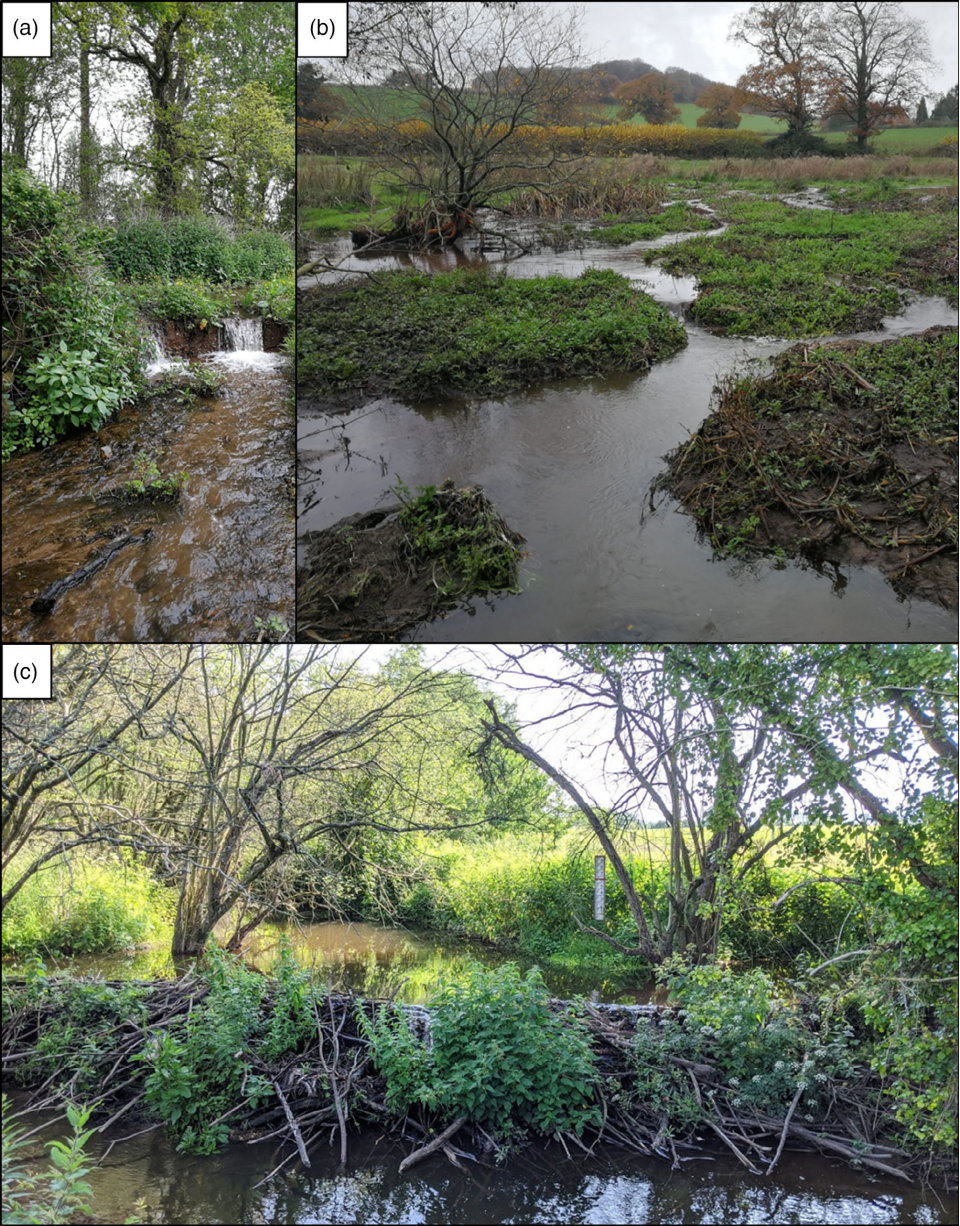
Examples of dam construction and channel avulsion resulting from beaver dam construction from the River Otter catchment, England. Panel (a) shows an example where a divergent flow path has re‐entered the main channel resulting in head‐cut erosion. Panel (b) shows the type of multi‐thread channel form that occurs downstream of dams in wide, low gradient floodplains. Panel (c) shows a beaver dam on a 4th order stretch of river. (Reproduced with permission from Photos © Hugh Graham and Alan Puttock)

Beaver dams are also key sites for channel avulsion (Giriat, Gorczyca, & Sobucki, 2016; John & Klein, 2004), as shown in Figure 1. John and Klein's (2004) study investigated the geomorphic impacts of beaver dams on the upland valley floor of the third‐order River Jossa (Spessart/Germany). Due to the creation of valley‐wide dams, which extended beyond the confines of the bank, multi‐thread channel networks developed across the floodplain. Newly created channels would deviate from the main stream channel, re‐entering the river some way downstream. At the point where the newly created channel enters the stream, a difference in elevation results in the development of a knickpoint. This knickpoint then propagates upstream through head‐cut erosion, eventually relocating the main stem of the channel.

#### 2.1.6 Agents of aggradation

Hydrogeomorphic changes, due to beaver engineering, are likely to have implications for stores and downstream fluxes of sediment and associated nutrients (Butler & Malanson, 1994; Lizarralde, Deferrari, Alvarez, & Escobar, 1996). Sediments mobilized and transported from upstream are deposited in beaver ponds, due to a decrease in velocity associated with a reduction in water surface gradient (Giriat et al., 2016) and consequently stream power (Butler & Malanson, 1994).

Pollock, Lewallen, Woodruff, Jordan, and Castro (2017) showed lower concentrations and loads of suspended sediment leaving a beaver site in contrast to those entering the site, while Puttock et al. (2018) showed that within the same site the beaver pond sequence was storing 100 t of sediment combined with an associated 16 t of carbon and 1 t of nitrogen. It is therefore suggested that beaver dams and ponds can create landscapes with depositional sediment regimes exerting a significant influence over channel sediment budgets, akin to the pre‐anthropocene dam and woody debris that once played a vital role in the evolution of river networks and floodplains, through the storage of sediment and nutrients and creation of riparian wetland and woodland (Brown et al., 2018).

The large mass of sediment (over 70 kg per m^2^ of ponded extent) being stored in a relatively small area (1.8 ha) reported by Puttock et al. (2018) represents similar levels of aggradation to those reported in studies, primarily from North America. Beaver dam sequences on low order streams have previously been shown to account for up to 87% of sediment storage at reach scales, while the removal of a sequence of beaver dams in Sandon Creek, British Colombia, leads to the mobilization of 648 m^3^ of stored sediment (Butler & Malanson, 1994, 1995; Page et al., 2005). Butler and Malanson (1994, 1995), also reported sediment accumulation rates of 2–28 and 4–39 cm year^−1^ for different beaver pond sequences in Glacier National Park, Montana. Values of sediment accumulation from North American beaver systems indicate the estimated average accumulation value of 5.4 cm year^−1^ presented by Puttock et al. (2018) in Great Britain may be at the lower end of what is possible in bigger dam–pond complexes or systems with a more plentiful sediment supply. In one of the few other studies in European landscapes, de Visscher et al. (2014) studied sediment accumulation in two beaver pond sequences in the Chevral River, Belgium. de Visscher et al. (2014) estimated the total sediment mass deposited in the dam sequences at 495.9 t. From the two pond sequences, average pond area was 200.4 m^2^, average sediment depth 25.1 cm, and average sediment mass of 14.6 t, equating to a normalized mass of 72.65 kg of sediment deposited per m^2^ of the pond. These values are very similar to the mean sediment depth of 27 cm and mean normalized mass of 71.40 kg m^2^ reported from the intensively managed grassland catchment in the UK (Puttock et al., 2018).

The sediment data published also demonstrate that beaver ponds can exhibit high sediment accumulation rates in comparison with other wetland systems. As an example, in a review of sediment accumulation rates in freshwater wetlands (Johnston, 1991) a mean annual accumulation rate of 0.69 cm year^−1^ was reported across 37 different wetland types, ranging from riparian forest to wet meadows. As with the biodiversity benefits of beaver ponds (see Willby et al., 2018 and Section 3 below) the high sediment accumulation rate of beaver ponds in relation to other freshwater wetlands, may reflect the highly dynamic nature of beaver systems, their constant evolution, and sustained maintenance (i.e., continuous dam‐building).

The long‐term fate of sediment will depend on the availability and composition of deposited sediment, the flow regime, and the preservation of dam structures (Butler & Malanson, 2005; de Visscher et al., 2014). Over many years, sediment may continue to accumulate until each pond fills completely and sediments are colonized by plants forming beaver meadows (Polvi & Wohl, 2012). However, beavers can also contribute to downstream sediment budgets; through the excavation of canal networks and bank burrows (de Visscher et al., 2014; Lamsodis & Ulevičius, 2012), in addition to the release of sediment following dam outburst floods (Curran & Cannatelli, 2014; Levine & Meyer, 2014). Beaver dam failure can result in releases of sediment (Polvi & Wohl, 2012) meaning that sediment storage in ponds can be transient (de Visscher et al., 2014). However, different sediment retention dynamics have been reported following dam collapse. For example, Giriat et al. (2016) found that there were very minimal losses of sediment from beaver ponds studied in Poland, following a dam collapse. Similarly, the majority of sediments were retained in ponds and subsequently stabilized following dam reconstruction (Curran & Cannatelli, 2014; Levine & Meyer, 2014) most likely reducing the downstream release of sediment from any single dam failure within the complex (Butler & Malanson, 2005; Puttock et al., 2018). While recent studies in North America involving extensive survey work have expanded knowledge of beaver dam persistence significantly (Hafen et al., 2020), including persistence during large rainstorm events (Westbrook, Ronnquist, & Bedard‐Haughn, 2020), resilience, failure, and associated sediment dynamics are likely to be highly spatially and temporally variable. As identified in Section 2.2 for both hydrological, geomorphic, and associated sediment/water quality impacts a greater mechanistic understanding of dam failure is therefore still required.

Finally, high levels of nutrient‐rich sediment have also been shown to result in further biogeomorphic alterations, that is, colonization by homogeneous patches of herbaceous or shrubby species, adding roughness to topography, reduced water velocities, and encouraging further deposition of sediments. Additionally, partial felling and submergence of woody debris disrupts flows and when felled in‐channel, creates reinforcement for existing dam structures (Curran & Cannatelli, 2014).

#### 2.1.7 Impacts of dams on river profile

Beaver dams have two main effects on river profile; (a) long‐profile is altered such that a stepped profile develops with sections of reduced gradient, that promote aggradation, upstream of dams separated by hydraulic jumps, created by flow over the dams, which initiates erosion. (b) Channel planform typically increases in complexity with many studies reporting; greater sinuosity, channel width, and the development of a multi‐thread planform (Ives, 1942; John & Klein, 2004; Pollock et al., 2014; Wegener, Covino & Wohl, 2017). These increases in cross‐profile complexity are driven by an increase in the heterogeneity of flow direction, which drives lateral flow, increasing bank erosion, channel widening, and subsequent localized deposition (Gorczyca et al., 2018).

#### 2.1.8 Agents of river restoration

In an undisturbed or near‐pristine riverine system, the engineering behavior of beaver may simply maintain an evolving geomorphic structure, sustaining a state of dynamic equilibrium in river function. In degraded landscapes (which are much more common), where river planforms are incised, single thread, straightened, even dredged, and lacking in geomorphic diversity, beaver have a dramatic impact on channel planform at multiple scales. In North America, beaver dams and their human‐constructed counterparts, known as beaver dam analogs, have been shown to restore degraded river systems (Pollock, Beechie, & Jordan, 2007), primarily through the aggradation of channel beds, leading to greater channel‐floodplain connectivity (Macfarlane et al., 2015; Pollock et al., 2014).

**FIGURE 2:**
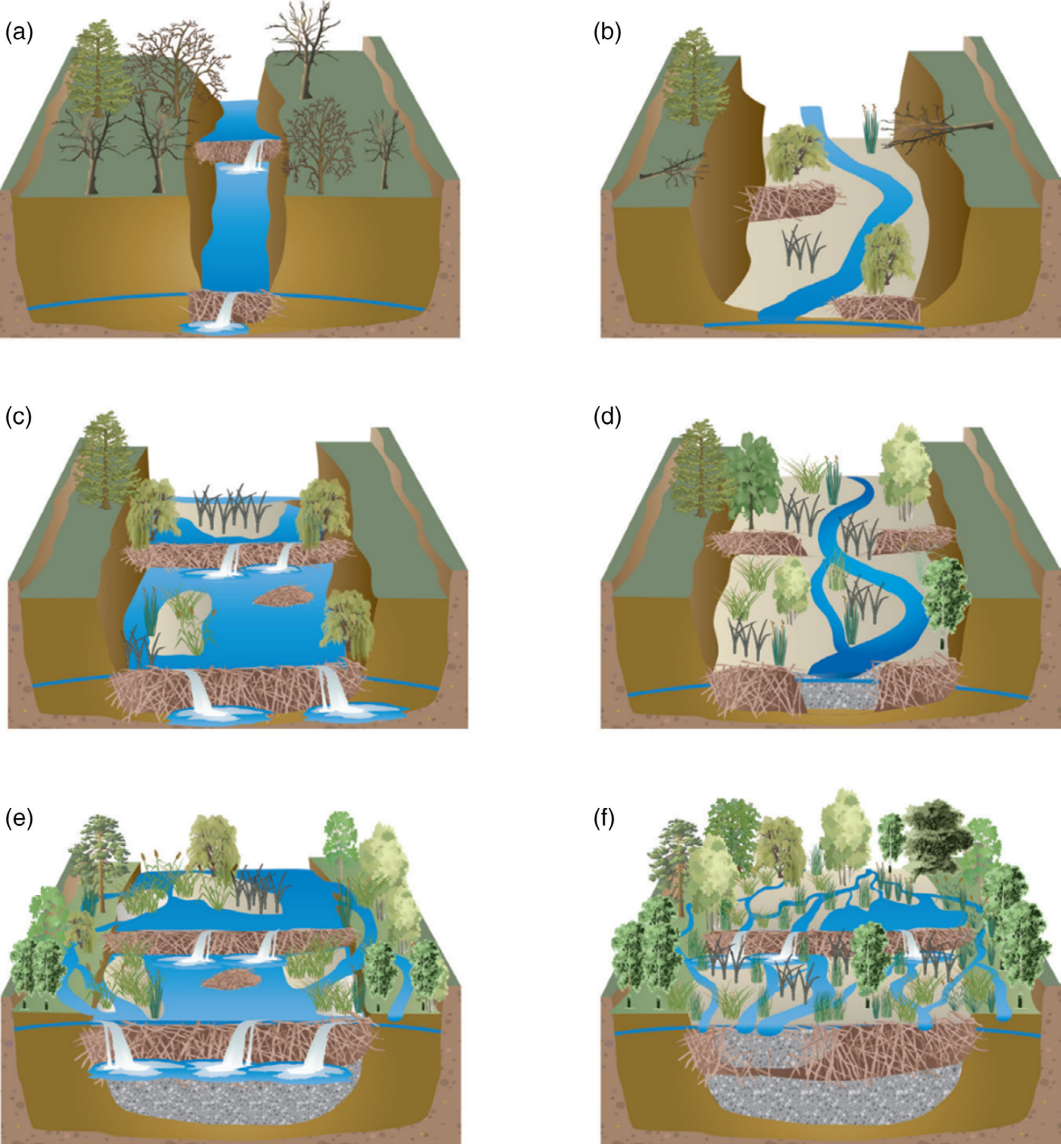
The influence of beaver activity on the geomorphology of incised streams: (a) low‐flow damming of confined channels with high‐flow blowouts causes overtopping, bank widening, and excavation of the channel bed; (b) sediment becomes more mobile and the channel reconfigures with vegetation establishment; (c) channel widening reduces high‐flow peak stream power and this provides suitable conditions for wider, more stable dams; (d) sediment accumulates in ponds and raises the height of the channel with dams overtopped and small blow‐outs occurring where dams are abandoned; (e) process repeats until dams are rebuilt, channel widens and the water table rises sufficiently to reconnect river channel to the floodplain; and (f) high heterogeneity occurs with vegetation and sediment communities establishing themselves, multi‐threaded channels and ponds increase reserves of surface water and dams and dead wood reduce flows and provide wetland habitats. (Reproduced with permission from Pollock et al., 2014)

Dams, however, are not rigid structures—they influence and are influenced by flow regimes (Johnston & Naiman, 1987) as is evidenced in Figure 2 (after Pollock et al., 2014). In narrow, incised channels, typical of degraded landscapes, beaver dams will capture some sediment but predominantly provide a foci for erosion. In these confined channels, unit stream power is high and therefore dams will frequently blow‐out and erode laterally. The resultant effect is a widening of the channel, which leads to a concomitant decline in stream power, thus allowing for greater aggradation rates and less frequent blow‐outs altering the sediment regime from net erosional to net depositional (Butler, 1995; Butler & Malanson, 2005). Over time, incised, straightened streams can be restored to complex multi‐threaded channel systems that represent a return to the pre‐anthropocene streams and rivers that were once common across north‐west Europe (Brown et al., 2018). In Poland, beaver initiated geomorphic processes were shown to alter artificially homogenized river reaches and thus it has been suggested that they may have a substantial role to play in the renaturalization of river systems (Gorczyca et al., 2018).

#### 2.1.9 Summary of geomorphic impacts

- Beaver damming activity is mostly limited to ≤fifth‐order streams as low stream power is favorable for dam‐building and persistence, with a reduction in the frequency of blowouts.
- Beavers drive a transition in sediment dynamics from dominantly erosional to net depositional, while increasing the spatial variability of both erosional and depositional features.
- Geomorphic change due to beaver is often characterized by changes in channel planform, longitudinal profiles, water surface and channel bed slope, increased sinuosity, and enhanced floodplain connectivity and surface roughness.

#### 2.1.10 Gaps in geomorphic understanding

- At present, the majority of geomorphology‐facing beaver research is from North America. Several studies from Europe indicate strong parallels between the geomorphic impacts between continents. However, geomorphic impacts are strongly influenced by local geography and therefore further monitoring is necessary to complement these findings.
- Research on the impacts of beaver on geomorphic processes is required at larger spatial extents and longer temporal scales. At present, most research focuses on site/reach scale observations, which must be continued in dialogue with long‐term, catchment scale monitoring and modeling to build understanding at landscape scales.
- The effects of beaver activity on short‐term sediment storage/mobilization due to bank‐burrowing and canal excavation, has not yet been substantially investigated.

### 2.2 Impacts of beaver upon hydrology

#### 2.2.1 Overview

There is an increased need to recognize the influence of biology upon river form and process (Johnson et al., 2020) and beavers as recognized ecosystem engineers are a key example of the ability of an animal to influence hydrological functioning. While other beaver engineered structures discussed in Section 2.1, such as burrows and canals, have a measurable impact (Grudzinski, Cummins, & Vang, 2019), the biggest (and most studied) hydrological impact of beavers results from their dam‐building ability and the consequent impoundment of large volumes of water in ponds (Butler & Malanson, 1995; Hood & Bayley, 2008). Dam and pond features can alter hydrological regimes, both locally and downstream (Burchsted & Daniels, 2014; Polvi & Wohl, 2012). Beaver activity can reduce downstream hydrological connectivity, and conversely increase lateral connectivity, forcing water sideways into neighboring riparian land, inundating floodplains, and creating diverse wetland environments (Macfarlane et al., 2015), while also contributing to soil and groundwater recharge (Westbrook et al., 2006).

Multiple studies have identified beaver dam sequences and wetlands as a cause of flow attenuation—so‐called “slowing the flow” (Green & Westbrook, 2009; Gurnell, 1998; Pollock et al., 2007). This impact has been attributed to the increase in water storage in beaver pond sequences, relative to undammed reaches (Westbrook et al., 2020), and increased hydrological roughness from the creation of dams and complex wetlands (Puttock et al., 2017), resulting in water being trapped or slowed as it moves through, over and around beaver dams. For example, Green and Westbrook (2009) found the removal of a sequence of beaver dams resulted in an 81% increase in flow velocity. The slow movement of water in beaver impacted sites is attributed to two main mechanisms: (a) increased water storage and (b) stream discontinuity and reduced longitudinal hydrological connectivity (Puttock et al., 2017). The increase in storage provided by beaver ponds and wetlands (Grygoruk & Nowak, 2014; Gurnell, 1998; Woo & Waddington, 1990) lengthens water retention times and reduces the velocity of the water. This in turn can increase the duration of the rising limb of the flood hydrograph which can reduce the peak discharge of floods (Burns & McDonnell, 1998; Green & Westbrook, 2009; Nyssen, Pontzeele, & Billi, 2011). Additionally, water stored in beaver ponds is released slowly as the porous dams gently leak both during and following rainfall, elevating stream base flows even during prolonged dry periods (Majerova, Neilson, Schmadel, Wheaton, & Snow, 2015; Puttock et al., 2017; Woo & Waddington, 1990), increasing environmental resilience to risks including drought and fire (Fairfax & Whittle, 2020).

Water levels in ponds vary significantly as a result of meteorological conditions both over long (i.e., seasonal) and short (i.e., inter‐event) timeframes (Puttock et al., 2017; Westbrook et al., 2020). Consequently, seasonal variations in water storage have been observed (see Majerova et al., 2015 for example). It might be expected that the attenuating impact of flow due to storage will be less during wet periods. However, it has been proven that beaver activity still attenuates flow during large events. For example, see Nyssen et al. (2011) who conducted one of the few in‐channel hydrological studies of Eurasian beaver; finding that flow attenuation was in fact greatest during largest events. In 2013, Westbrook et al. (2020) monitored the largest recorded flood in the Canadian Rocky Mountains west of Calgary, Alberta, challenging the commonly held assumption that dams fail during large floods (the majority fully or partially persisted) and showing that water storage offered by beaver dams (even failed ones) delayed downstream flood peaks. Therefore, it has been argued that the observed discontinuity or reduced downstream hydrological connectivity resulting from beaver dam‐building activity—also shown by Butler and Malanson (2005), is a key reason for the flow attenuation impact persisting even for larger events during wetter periods (Puttock et al., 2017).

Of course, beaver dam construction is highly variable and depends on the existing habitat, building material availability, and channel characteristics (Collen & Gibson, 2000; Woo & Waddington, 1990). Woo and Waddington (1990) identified multiple ways in which dam structure will influence flow pathways and that streamflow can overtop or funnel through gaps in the dams, leak from the bottom of the dams or seep through the entire structure. While the impact of dam structure upon connectivity and therefore, flow velocity will differ (Hering et al., 2001; Woo & Waddington, 1990), all dams will increase channel/hydraulic roughness and therefore, deliver some flow attenuation effect, which can be most significant when a suite of dams in close proximity are constructed (for example see Puttock et al., 2017 case study). Thus, in addition to dam structural variations, it is important to note that the number of dams and their density will strongly influence any observed differences in hydrological function. Existing work has also discussed the importance of the number of dams in a reach, with beaver dams having the greatest impact on hydrology when they occur in a series (Beedle, 1991; Gurnell, 1998). Similarly, sequences of (non‐beaver) debris dams in third order, Northern Indiana (USA) streams were found to increase the retention time of water by a factor of 1.5–1.7 (Ehrman & Lamberti, 1992). Ponds located in series provide both greater storage and greater roughness, resulting in a greater reduction in flow velocities as shown by Green and Westbrook (2009). In another study, pond sequences have been shown to reduce the peak flows of 2‐year return floods by 14% whereas individual dams reduced flood peaks of similar events by only 5.3% (Beedle, 1991).

There are very few hydrological modeling studies into the impacts of beaver dam sequences upon flow regimes. In European landscapes, this perhaps reflects the fact that until recently there has been both a dearth of beaver dams themselves and also a lack of empirical understanding of the impact on hydrological functioning. In a notable exception, Neumayer, Teschemacher, Schloemer, Zahner, and Rieger (2020) undertook hydraulic modeling of beaver dam sequences and evaluated their impacts during flood events. Utilizing surveys of beaver dam cascades in Bavaria and 2D hydraulic modeling, Neumayer et al. (2020) predicted that during small flood events, beaver dams can deliver significant impacts upon peak flows (up to 13% reductions) and lag/translation times (up to 2.75 hr). But, Neumayer et al. (2020) also predicted that during larger floods (return period ≥2 years), the impact upon peak flows of a single dam sequence may be smaller (ca. 2%) and perhaps negligible at the catchment outlet. However, Neumayer et al. (2020) modeled the impacts of beaver dams on channels larger than those that other research has shown might support the greatest densities of dams (i.e., Graham et al., 2020 show that dams rarely persist on >fifth‐order streams) and thus it is suggested that further modeling work is required into the downstream hydrological impacts of small streams with high dam densities. In addition, further research is required to understand what the cumulative catchment outlet effects might be if beavers return to being widespread and catchments contain multiple dam sequences (i.e., hundreds of dams) in all headwater streams.

#### 2.2.2 Summary of hydrological impacts

- Beavers can reduce longitudinal (downstream) connectivity, while simultaneously increasing lateral connectivity, pushing water sideways.
- Beavers can increase surface water storage within ponds and canals, while also elevating the water table and contributing to groundwater recharge.
- Beaver dam sequences and wetlands can attenuate flow during both high and low flow periods.

#### 2.2.3 Gaps in understanding: Hydrology

- A greater mechanistic understanding of the hydrological impacts of beaver dams and also critically sequences of beaver dams across scales and land uses to inform hydrological modeling, management, and policy decision making.
- Conditions of dam failure and consequences.
- Greater understanding of beaver landscape engineering upon low flow conditions and wetland maintenance during drought.

### 2.3 Impacts of beaver upon water quality

The altered flow regimes and water storage capacity discussed in Section 2.2 can also modify sediment regimes and nutrient and chemical cycling in freshwater systems. As a consequence of reduced downstream connectivity and a change from lotic to lentic systems, beaver activity is believed to alter both local and downstream sediment dynamics, and water quality via both abiotic and biotic processes (Cirmo & Driscoll, 1996; Johnston, Pinay, Arens, & Naiman, 1995). It has been argued that two key mechanisms affect the difference in sediment dynamics of water quality observed in beaver systems: (a) slowing of flow resulting in the physical deposition of sediment (reviewed in Section 2.1) and associated nutrients/chemicals, (b) an increase in both ponded water and a local rise in water tables, results in an overall increase in wetness altering the biogeochemical cycling of nutrients (Puttock et al., 2017).

#### 2.3.1 Impacts on nutrient cycling

When beaver dams inhibit the transport of fine sediments, large volumes of organic and inorganic compounds become stored within beaver ponds (Rosell et al., 2005), including; nitrogen, phosphorus, and particulate (bound) carbon (Lizarralde et al., 1996; Naiman, Pinay, Johnston, & Pastor, 1994). This change increases the volume of anoxic sediments and provides organic material to aid microbial respiration. Nutrients are temporarily immobilized in pond sediments and taken up by aquatic plants, periphyton, and phytoplankton. Increases in plant‐available nitrogen, phosphorus, carbon, and increased light availability (due to canopy reduction) favor the growth of instream and riparian vegetation, thus further immobilizing nutrients within plant biomass that re‐establishes local nutrient cycles (Rosell et al., 2005). In addition to the impacts of large volumes of sediment, the reduction in free‐flowing water and increased decomposition has been shown to increase anaerobic conditions in both pond surface water and saturated soils (Ecke et al., 2017; Rozhkova‐Timina, Popkov, Mitchell, & Kirpotin, 2018).

Lazar et al. (2015) show that beaver ponds have a denitrification impact while results from Puttock et al. (2017) showed Total Oxidized Nitrogen (TON) and Phosphate (PO_4_‐P) to be significantly lower in waters leaving a beaver impacted site compared with water quality entering. These reductions manifest both in terms of concentrations and loads of nutrients, suggesting that beaver activity at the site created conditions for the removal of diffuse pollutants from farmland upstream. Correll, Jordan, and Weller (2000) found that prior to dam construction, TON concentrations were significantly correlated with river discharge but after dam construction, no significant relationship was observed, although there was a correlation between discharge and nitrate (NO_3_‐N). Similarly, Maret, Parker, and Fannin (1987) identified reductions in Total Kjeldahl Nitrogen (TKN) downstream of beaver dams during high flows. It has also been shown that beaver ponds are particularly effective at NO_3_‐N retention (K. J. Devito, Dillon, & Lazerte, 1989). It is suggested, therefore, that in agriculturally dominated catchments where diffuse pollution rates are high, beaver ponds may be effective tools to manage N‐related diffuse pollution problems from intensive agriculture upstream (Lazar et al., 2015).

Puttock et al. (2017) show that beaver ponds can also act as sinks for phosphorus associated with sediments, while Maret et al. (1987) identified that suspended sediment was the primary source of phosphorus found leaving a beaver pond; therefore, during conditions when more sediment is retained behind the dam than is released, total phosphorus retention will increase. In a study of a beaver impacted and non‐beaver impacted catchment (Dillon, Molot, & Scheider, 1991), found total phosphorus export was higher in the non‐impacted catchment suggesting that phosphorus was being stored somewhere within the catchment—most probably in the beaver ponds. Lizarralde et al. (1996) also reported that while phosphorus concentrations were significantly higher in riffle sediments, due to extensive wetland creation, total storage was highest in Patagonian beaver ponds. Previous studies have focused primarily on the relationship between discharge and phosphorus concentrations and yields leaving ponds, with inconclusive results. Devito et al. (1989) reported a strong positive correlation between phosphorus loads and stream discharge. However, Maret et al. (1987) report a negative correlation between phosphorus concentrations and discharge and Correll et al. (2000) report no correlation between nutrient flushing and stream discharge following dam construction. Climatic and seasonal changes (Devito & Dillon, 1993; Klotz, 2007) and organic matter availability (Klotz, 2007, 2013) have been shown to affect in‐pond phosphorus‐dynamics. With regard to downstream impact, the key consensus, that is supported by the correlation between suspended sediment and phosphate concentrations observed in Puttock et al. (2017) is that beaver ponds are effective at retaining phosphorus associated with high sediment loads (Devito et al., 1989; Maret et al., 1987).

Ecke et al. (2017) suggest age dependency as a factor in nitrogen and phosphorus dynamics, with older, more solid dams increasing retention compared to younger more leaky dams. In a review of beaver impacts upon nitrogen and phosphorus content in ponds and downstream, Rozhkova‐Timina et al. (2018) cite contradictory information and study results as showing there is a strong contextual dependence and it is clear that further research into the controlling mechanisms of nutrient retention is required.

In contrast to the trends observed for nitrogen and phosphorus, multiple studies, that is, Puttock et al. (2017) and Cazzolla Gatti et al. (2018) found concentrations and loads of Dissolved Organic Carbon (DOC) increase due to beaver activity. This increase is attributed to enhanced sediment and nutrient storage in addition to the overall increase in wetland extent creating an environment rich in organic matter, as previously shown by Vecherskiy, Korotaeva, Kostina, Dobrovol'skaya, and Umarov (2011). Similarly, Law, McLean, and Willby (2016), using color as a proxy for DOC, observed increased concentrations below a series of beaver dams. Dams trap sediment‐bound particulate carbon meaning that ponds can act as net stores of carbon (D. Correll et al., 2000; Lizarralde et al., 1996; Naiman, Melillo, & Hobbie, 1986). However, as a consequence of this overall increase in carbon availability, significant exports of DOC have been observed either downstream (D. Correll et al., 2000; Naiman et al., 1994) or in comparison with non‐beaver impacted catchments (Błȩdzki, Bubier, Moulton, & Kyker‐Snowman, 2011). Several authors have speculated that the cause of this DOC release relates to (a) incomplete decomposition processes making DOC more available for loss (Cirmo & Driscoll, 1996); (b) enhanced production during primary productivity; (c) a product of enhanced microbial respiration (D. Correll et al., 2000); and (d) retention of particulate organic carbon and litter entering the site and subsequent decomposition (Law et al., 2016). Based upon research in western Siberia, Cazzolla Gatti et al. (2018) argue that beaver activity simultaneously increases nutrient cycling and DOC availability at the same time as increasing carbon sequestration as carbon is accumulated in sediment and removed from the short‐term carbon cycle.

pH has been shown to be a first‐order control on DOC production and transport in other wetlands (Clark, Lane, Chapman, & Adamson, 2007; Grand‐Clement et al., 2014). However, Cirmo and Driscoll (1996) found that a beaver impacted catchment contained higher levels of DOC both before and after CaCO_3_ treatment (to reduce acidity) when compared with a non‐impacted catchment, suggesting that pH plays a limited role in the production of DOC in beaver ponds. Puttock et al. (2017) showed pH to be marginally more alkaline in water leaving the site, which is in agreement with other studies showing more acidic waters in beaver ponds than immediately downstream (Cirmo & Driscoll, 1993; Cirmo & Driscoll, 1996; Margolis, Castro, & Raesly, 2001). However, whether these changes in pH were of a large enough magnitude to alter within site biogeochemical cycling is as yet unclear.

Increased water availability in beaver systems, in addition to a change in chemistry associated with a transformation from lotic to lentic waters, has also been ascribed by multiple studies to control increased leaching of heavy metals from soils and increased concentrations in waters downstream. Releases from pond or increases in downstream concentrations of calcium, iron, and magnesium (for example) were observed by Naiman et al. (1994) and C. A. Johnston et al. (1995), while Levanoni et al. (2015) and Margolis et al. (2001) also observed downstream increases in manganese and observed increasing methylmercury concentrations both downstream of beaver sites and in macroinvertebrates within beaver sites. In a meta‐analysis review, Ecke et al. (2017) found young ponds to be a source for methylmercury in water, while old ponds were not, again highlighting that beaver systems are complex and dynamic with a high degree of context‐dependence required to understand their impacts upon water quality.

#### 2.3.2 Summary of water quality impacts

- Beaver wetlands and dam sequences can change parts of freshwater ecosystems from lotic to lentic systems impacting upon sediment regimes and biogeochemical cycling.
- By slowing the flow of water, suspended sediment and associated nutrients are deposited, with ponds shown to be large sediment and nutrient stores.
- Increased water availability, raised water tables, and increased interaction with aquatic and riparian vegetation have all been shown to impact positively upon biogeochemical cycling and nutrient fluxes.

#### 2.3.3 Water quality gaps in understanding

- Sediment and nutrient dynamics within dam sequences as opposed to individual dams and ponds.
- A greater understanding is required of the contributing source of sediment and nutrients to beaver ponds.
- How long‐term beaver dam sequences and wetland dynamics contribute to downstream water quality.
- How the impoundment of water, sediments, and associated nutrients in ponds affects biogeochemical cycling and resulting transfers of nutrients in both gaseous and dissolved forms to understand the contribution of beavers to overall nutrient budgets in both the carbon and nitrogen cycles.

## 3 BEAVER IMPACTS UPON LIFE—CONTEMPORARY UNDERSTANDING

### 3.1 Impacts of beaver upon aquatic ecology

Enhancement of natural processes, floodplain inundation, lateral connectivity, and structural heterogeneity in beaver‐impacted environments creates a diverse mosaic of habitats. Such habitats are underpinned by greater provision of food, refuge, and colonizable niches, which form the cornerstone of species‐rich and more biodiverse freshwater wetland ecosystems (Brazier et al., 2020; Campbell‐Palmer et al., 2016; Gaywood et al., 2015; Gurnell, 1998; Rosell et al., 2005; Stringer & Gaywood, 2016). Readers are directed to three reviews on this topic: Stringer and Gaywood (2016), which provides a comprehensive overview of the impacts of beaver on multiple species, Dalbeck et al. (2020) which considers the impacts of beavers on amphibians in temperate European environments and Kemp, Worthington, Langford, Tree, and Gaywood (2012) which provides a valuable meta‐analysis of the impacts of beaver on fish. This section builds on these reviews to summarize the findings of research into the impacts of beaver activity on aquatic plants, invertebrates, and fish. We focus on these groups as they are widely considered to be strong indicator species of freshwater health and function (Herman & Nejadhashemi, 2015; Law et al., 2019; Turley et al., 2016).

#### 3.1.1 Aquatic vegetation (macrophytes)

Beavers affect aquatic vegetation through direct and indirect mechanisms over a range of spatial and temporal scales (Rosell et al., 2005). Natural disturbances, including; herbivory, food caching, tree‐felling (Campbell‐Palmer et al., 2016; Harrington, Feber, Raynor, & Macdonald, 2015), and/or dam‐induced extension of wetland area (Gurnell, 1998; Puttock et al., 2017) can aid macrophyte recruitment (Levine & Meyer, 2019), regenerate riparian areas (Jones, Gilvear, Willby, & Gaywood, 2009), and enhance plant biodiversity from the local to the landscape scale (Law, Bunnefeld, & Willby, 2014; Law, Jones, & Willby, 2014; Law, Levanoni, Foster, Ecke, & Willby, 2019; Willby et al., 2018). Canopy‐opening and floodplain inundation creates wetland areas with reduced shading (Donkor & Fryxell, 2000; Johnston & Naiman, 1990), providing opportunities for shade‐intolerant, opportunistic, and wetland plant species (Law et al., 2016, 2017; Law, Levanoni, et al., 2019; Marshall, Hobbs, & Cooper, 2013). Early successional shifts in newly created wetted zones promote emergent vegetation (Ray, Rebertus, & Ray, 2001), while transitional edges form around pond margins, characterized by rich, diverse, and structurally complex plant communities (McMaster & McMaster, 2001).

Over time, beaver wetland creation, maturation, and abandonment, can result in the siltation of ponds, creating novel habitats in marshy beaver meadows characterized by spatial variability in moisture‐regimes which drives higher plant species richness (Polvi & Wohl, 2012; Ray et al., 2001; Wright, Flecker, & Jones, 2003; Wright, Jones, & Flecker, 2002). As beaver meadows mature, terrestrial succession often occurs, leading to herbaceous encroachment, typically comprising grasses, shrubs, and sedges, with studies showing evidence of an eventual return to open, forested, stream environments (Johnston, 2017; Little, Guntenspergen, & Allen, 2012; McMaster & McMaster, 2001; Naiman, Johnston, & Kelley, 1988; Pollock et al., 1995; Ray et al., 2001).

#### 3.1.2 Invertebrates and amphibians

Beaver increase the heterogeneity of stream depth, flow velocity, and benthic habitats such as silty substrates, woody material (Clifford, Wiley, & Casey, 1993; France, 1997; Rolauffs, Hering, & Lohse, 2001), and both submerged and emergent vegetation, which separately support unique invertebrate species and assemblages (Benke, Ward, & Richardson, 1999; Bush & Wissinger, 2016; Law, Levanoni, et al., 2019; Wissinger & Gallagher, 1999). Beaver ponds support more lentic species (Collen & Gibson, 2000; Margolis et al., 2001; Rosell et al., 2005) and typically demonstrate increased invertebrate abundance (Czerniawski & Sługocki, 2018; Osipov, Bashinskiy, & Podshivalina, 2018; Strzelec, Białek, & Spyra, 2018; Willby et al., 2018), biomass (Osipov et al., 2018) and/or density (McDowell & Naiman, 1986). Beaver ponds may harbor unique assemblages, dominated by collector‐gatherers, shredders, and/or predators (Law et al., 2016; McDowell & Naiman, 1986; Robinson, Schweizer, Larsen, Schubert, & Siebers, 2020; Strzelec et al., 2018). However, diversity may be reduced due to the typically homogeneous benthic habitat within ponds resulting from increased fine sediment deposition (Descloux, Datry, & Usseglio‐Polatera, 2014; Pulley, Goubet, Moser, Browning, & Collins, 2019). At broader scales, varying successional stages in beaver wetlands, as well as longitudinal variability in habitat type along with beaver dam‐pond sequences (e.g., Margolis et al., 2001), increases the taxonomic, trophic, and/or β‐diversity of aquatic invertebrate communities compared to environments lacking beaver modification. This is primarily due to the heterogeneity of habitat benefiting a range of both lotic and lentic species (Bush, Stenert, Maltchik, & Batzer, 2019; Law et al., 2016; Pollock et al., 2017; Willby et al., 2018). Furthermore, the storage of sediment and nutrients within beaver ponds improves water quality (Puttock et al., 2017) downstream and therefore enhances habitat for pollution‐sensitive species (Rosell et al., 2005; Strzelec et al., 2018).

The gradual release of water from beaver ponds maintains flows during dry periods (Section 2.1), thereby increasing invertebrate resilience to drought by providing refuge pools and greater post‐drought recolonization potential (Wild, 2011; Wissinger & Gallagher, 1999). High‐head dams promote high velocity and turbulent water over, through, or around dams in side‐channels, creating habitat suitable for lotic species, which can otherwise be rare in low‐gradient stream reaches (Clifford et al., 1993; Law et al., 2016). In addition, cold hyporheic upwelling and lower stream temperatures downstream of high‐head dams, and at depth in beaver ponds, has been shown to benefit the reproductive success of invertebrate species such as mayflies (Fuller & Peckarsky, 2011).

Beaver‐engineered woody structures, such as dams and lodges, offer key invertebrate habitats resulting in greater abundance (France, 1997), biomass, density (McDowell & Naiman, 1986; Rolauffs et al., 2001), productivity, richness (France, 1997; Rolauffs et al., 2001), and diversity (Benke, Van Arsdall, Gillespie, & Parrish, 1984) compared to beaver ponds and free‐flowing streams. Direct benefits for invertebrates arise from physical complexity, such as the interstices of dams, lodges, bank burrows, and canals, which offer spaces suitable for novel microhabitats (Hood & Larson, 2015; Willby et al., 2018), refuge from predators (Benke & Wallace, 2003), egg‐laying (oviposition) sites (Gaywood et al., 2015), and emergent metamorphosis (Wallace, Grubaugh, & Whiles, 1993). These woody structures also provide attachment sites for filter‐feeding organisms and foraging resources for species that feed on woody material (xylophagous) and those that feed on the epixylic biofilms which grow on woody surfaces (Godfrey, 2003; Hering et al., 2001; Strzelec et al., 2018). For example, deadwood‐eating (saproxylic) beetles are known to occupy beaver‐impacted habitats (Horák, Vávrová, & Chobot, 2010; Stringer & Gaywood, 2016). In addition, the retention of organic particulate matter in beaver ponds enhances foraging opportunities for aquatic invertebrates, particularly gatherers and shredders (Johnston, 2014; Law et al., 2016; Wohl, 2013). Organic drift can also bring wider benefits within catchments, increasing the abundance and/or richness of invertebrates in areas both downstream (Redin & Sjöberg, 2013) and upstream (Rolauffs et al., 2001) of beaver‐modified sites.

Dalbeck et al. (2020) conclude that beavers and their habitat creating activities can be pivotal determinants of amphibian species richness, particularly in the headwater streams. The creation of lentic zones in beaver modified wetlands is cited as an essential breeding habitat for amphibian species, but can also be important for entire life history requirements (Cunningham, Calhoun, & Glanz, 2007), with beaver ponds offering sites where reliable spawning and early metamorphosis can take place, in instances comprising exclusive ovipositional sites within wider wetlands (Dalbeck, Janssen, & Luise Völsgen, 2014). Beaver modifications, which increase lentic‐rich habitat heterogeneity and/or raise light levels and solar radiation, warming patches of water, in turn, support healthier amphibian assemblages. Such improvements manifest via greater species‐richness (Cunningham et al., 2007), diversity (Bashinskiy, 2014; Cunningham et al., 2007; Dalbeck, Lüscher, & Ohlhoff, 2007), colonization rates and abundance (Anderson, Paszkowski, & Hood, 2015; Dalbeck et al., 2014; Stevens, Paszkowski, & Foote, 2007), older‐pond density (Stevens et al., 2007), size and productivity compared to unmodified habitats, with connectivity between ponds and through beaver canals reducing distances between breeding and foraging sites (Anderson et al., 2015). Woody complexes which form lodges and dams may also provide valuable habitat which amphibians can use for larval food provision and development (Tockner, Klaus, Baumgartner, & Ward, 2006), potential overwintering hibernation sites (Stevens et al., 2007) or cover from predators (Tockner et al., 2006), with cover options offering predatorial and larval protection by areas of shallow emergent‐vegetated pond margins (Dalbeck et al., 2007; Vehkaoja & Nummi, 2015). Conversely, lotic obligate species may be negatively affected by beaver activity (Stringer & Gaywood, 2016), although studies have demonstrated the persistence and high abundance of stream‐dependent species on the unimpounded reaches of beaver modified streams (e.g., Cunningham et al., 2007).

#### 3.1.3 Fish

**FIGURE 3:**
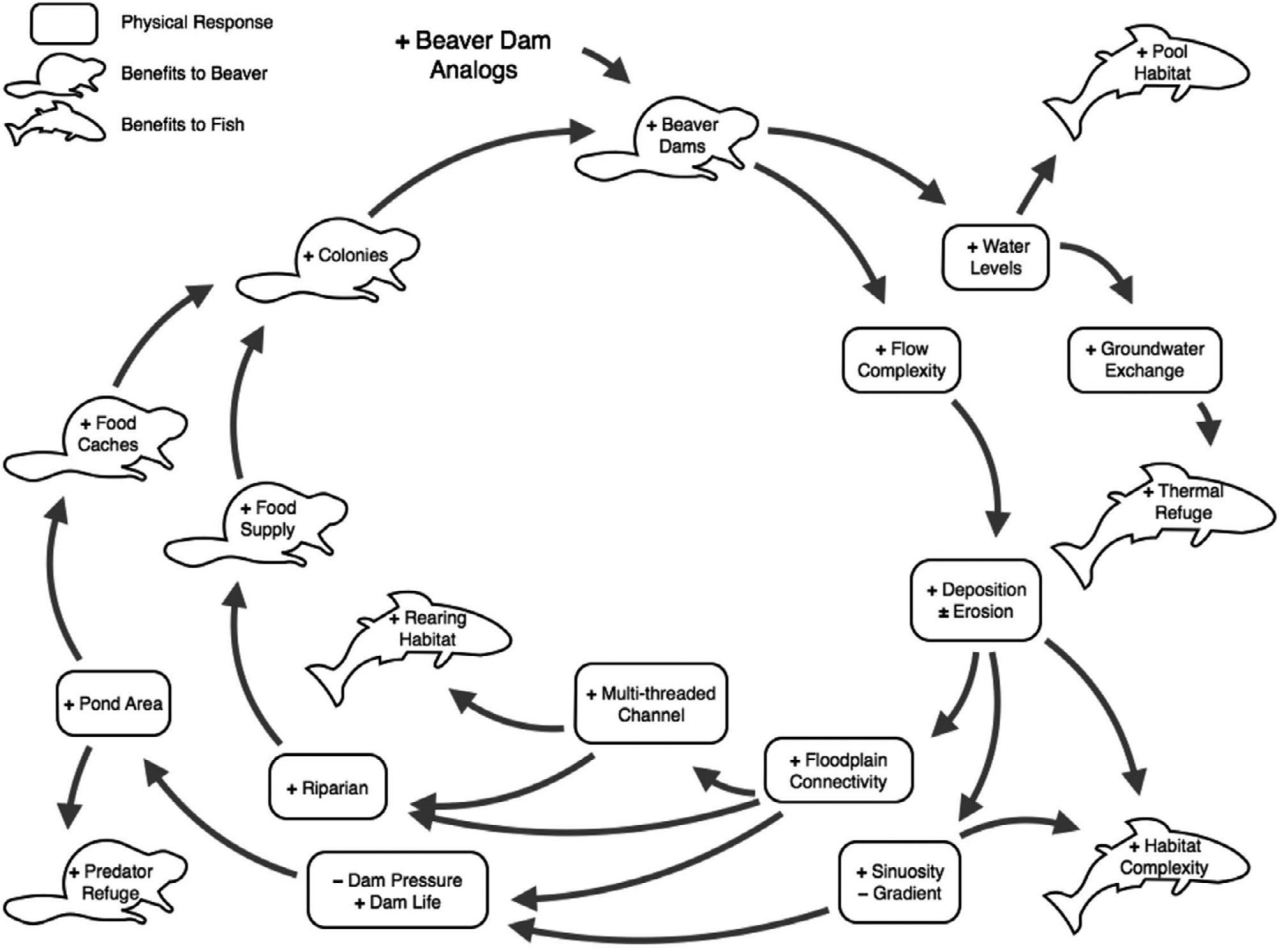
Flow Diagram of expected change following beaver return. (Reproduced with permission from Bouwes et al., 2016)

Beavers and fish have cohabited for millennia (Malison & Halley, 2020) and have previously been shown to coexist positively (Kemp et al., 2012). As such, it is no surprise that beaver‐induced habitat changes, particularly increased heterogeneity, can benefit fish populations (Figure 3). Documented benefits include increased: growth rates (Malison, Eby, & Stanford, 2015; Pollock, Heim, & Werner, 2003; Rosell & Parker, 1996), survival (Bouwes et al., 2016), biomass (Bashinskiy & Osipov, 2016), density (Bouwes et al., 2016; Wathen et al., 2019), productivity (Osipov et al., 2018; Pollock et al., 2003; Pollock, Pess, Beechie, & Montgomery, 2004), species richness (Snodgrass & Meffe, 1998), and diversity (Smith & Mather, 2013). Additional benefits to fish include the creation of juvenile rearing habitat (Johnson & Weiss, 2006; Leidholt‐Bruner, Hibbs, & McComb, 1992; Pollock et al., 2004), overwintering habitat (Chisholm, Hubert, & Wesche, 1987; Cunjak, 1996; Malison et al., 2015), migratory respite (Virbickas, Stakėnas, & Steponėnas, 2015), enhanced spawning habitat (Bylak, Kukuła, & Mitka, 2014), greater invertebrate food availability (Rolauffs et al., 2001), and refugia from low‐flows (Hägglund & Sjöberg, 1999), high discharge (Bouwes et al., 2016), temperature extremes (Wathen et al., 2019), and predation (Bylak et al., 2014). It is for these reasons, that recent approaches in the US have used beaver reintroduction to enhance habitat in support of salmonid reintroduction and/or conservation (Bouwes et al., 2016).

Due to the wide range of changes that beavers bring about, the benefits listed above will likely manifest for a variety of freshwater fish species through a wider understanding of these impacts is required as most research has focused upon interactions between beaver and salmonid species. Salmonids, particularly anadromous species (migrating from the sea to spawn in rivers) hold significant financial, cultural, and recreational value from a fisheries perspective (Butler, Radford, Riddington, & Laughton, 2009). Unfortunately, for a variety of reasons, which have nothing to do with beavers, populations of salmonid populations in Europe are in decline, and the two most abundant native salmonids, the Atlantic salmon (*Salmo solar*) and the Brown/Sea trout (*S. trutta*) are under threat (Forseth et al., 2017). Research in the US has largely shown that beaver reintroduction aids the recovery of salmonid populations (e.g., Bouwes et al., 2016; Wathen et al., 2019); however, despite the long‐term coexistence of these species, the expansion and reintroduction of beavers across European landscapes, now substantially altered due to anthropogenic activity, has raised concerns regarding the potential impact that beaver activity may have on salmonid species (Malison & Halley, 2020).

Two recent studies have investigated the impacts of beaver on salmonid habitat and populations in upland streams (Bylak & Kukuła, 2018; Malison & Halley, 2020). Both of these studies report increased habitat patchiness and heterogeneity in river systems that are typically dominated by fast‐flowing habitat. Neither study found evidence to suggest that beaver dams prevented fish movement either upstream or downstream. However, Malison and Halley (2020) did find that the presence of beaver dams affected the frequency of movement between stream reaches, suggesting that either beaver dams may act to restrict daily home ranges of salmonids, or the increased local habitat complexity around beaver dams reduces the need for salmonids to travel greater distances. A conflicting finding of these studies is that of the use of ponds by salmonids. In agreement with numerous studies that found beaver ponds to provide valuable rearing habitat (Malison, Lorang, Whited, & Stanford, 2014; Weber et al., 2017) and habitat niches for different stages of salmonid life cycles (Bouwes et al., 2016; Wathen et al., 2019), Bylak and Kukuła (2018) observed that brown trout used different beaver‐created habitats throughout their life stages. However, Malison and Halley (2020) reported that they did not observe beaver ponds being used as salmon rearing habitat. Both studies report either no significant effect of beaver on fish populations (Malison & Halley, 2020) or a positive impact on the community composition and patch dynamics (Bylak & Kukuła, 2018).

Virbickas et al. (2015) studied the impacts of beaver on two lowland Lithuanian streams. Unlike, the studies from upland streams, Virbickas et al. (2015) found evidence to suggest that beaver dam sequences do restrict upstream movement of salmonids with reaches below and between ponds being used but no salmonids or redds (spawning sites) being observed upstream of beaver dam complexes. While the presence of beavers did enhance community evenness upstream of dams, this effect was attributed to the exclusion of salmonids, which typically dominated fish communities downstream of dams.

The scale of such studies should be considered carefully in the context of mobile and dynamic species of fish. Bylak and Kukuła (2018) present data from the longest period of monitoring in Europe. They show that the response of fish to beaver activity enhances metacommunity resilience but consequently localized fish communities may alter for short periods of time. However, in these upland systems, high flows capable of “blowing out” dams are more frequent (Macfarlane et al., 2017) thus allowing unimpeded fish movement during these periods. In lowland systems, such as those investigated by Virbickas et al. (2015) the increased hydrological stability may result in a longer lasting separation of fish communities up and downstream of beaver dams. In low gradient systems, where spawning habitat is located solely in the upper reaches of a catchment, the presence of dams could potentially limit access to these reaches, affecting spawning success or resulting in the formation of new spawning habitat, such as the clean gravel bars which commonly form at the tail end of beaver ponds and immediately downstream of dams (Bouwes et al., 2016).

Further research on the impacts of fish across varied European landscapes is required. These studies should seek to understand the effect of beaver on fish communities at the catchment scale. It is well established that fish can navigate beaver dams (Bouwes et al., 2016; Bylak & Kukuła, 2018; Malison & Halley, 2020; Virbickas et al., 2015). However, a greater understanding is required to quantify the importance of any reduced longitudinal movement of fish alongside the known benefits including an increase in food availability and greater habitat diversity.

#### 3.1.4 Aquatic ecology summary

- Beaver activity extending wetland areas aids aquatic plant recruitment, abundance, and species diversity.
- Nutrient‐rich beaver meadows result in mature beaver managed landscapes, contributing diverse plant life, and increasing patchiness in otherwise homogeneous (especially intensively farmed) landscapes.
- Heterogeneity of beaver habitat leads to greater diversity of invertebrates, benefitting both lotic, and lentic species.
- Slow release of water from beaver ponds elevates baseflow downstream supporting greater aquatic life, improving resilience especially in times of drought.
- A multitude of benefits accrue for fish due to beaver activity such as increased habitat heterogeneity and food availability.
- It is established that salmonid species can navigate beaver dams, though there is evidence that the presence of dams does alter the way they move within river networks. The impact of dams on salmonid movement is highly dependent on location and upstream movement may be reduced in low gradient, low energy systems.

#### 3.1.5 Aquatic ecology gaps in understanding

- Community level, catchment scale understanding of beaver interactions with fish of all species is required to determine whether the changes seen—returning freshwaters to something akin to pre‐anthropocene conditions, are overall positive (as current literature suggests) or negative and thus requiring management interventions.
- The narrow, riparian landscapes of many European countries, wherein intensive agriculture encroaches on freshwaters, need further research into the impacts of beavers on both existing vegetation and that which may emerge if more space for water and beavers is made.
- Changes to the ecological status of freshwaters inhabited by beavers are inevitable and research to understand the impact on goals of the Water Framework Directive is needed, to contextualize what is meant by “good” ecological status now that beavers are present.

### 3.2 Human–beaver interactions

The potential benefits and impacts of beaver reintroduction (outlined above for the environment) can also manifest for humans. Notably, flow attenuation resulting from beaver damming will be likely to reduce potential for flooding of properties downstream. There is a further socioeconomic benefit not as yet explored in this article; as beavers bring more wildlife to ecosystems, beaver lands can become a focus of wildlife tourism, where humans interact with wild animals or with animals in enclosures (Higginbottom, 2004; Moorhouse, D'Cruze, & Macdonald, 2017). Wildlife tourism is a growing global trend which can engage people with nature, with their experiences often contributing toward local communities, providing benefits for mental health and well‐being, and incentivizing nature conservation behaviors (Curtin, 2009; Curtin & Kragh, 2014; Higginbottom, 2004; Lackey et al., 2019; Newsome, Rodger, Pearce, & Chan, 2019; Skibins, Powell, & Hallo, 2013).

Much wildlife tourism is centered upon “charismatic species” (Curtin, 2010; Skibins et al., 2013), but some are motivated by the intention to support wider biodiversity rather than charismatic species alone (Hausmann, Slotow, Fraser, & Minin, 2017). Beavers are often considered charismatic and, as a keystone species, are associated with biodiverse landscapes, which they create and maintain. Thus, they exhibit both those traits that motivate wildlife tourism. Beaver tourism activities that currently exist in Europe include “beaver safaris”, guided tours of beaver‐modified landscapes, and information centers (Campbell, Dutton, & Hughes, 2007; Halley et al., 2012; Rosell & Pedersen, 1999). Beaver tourism and associated support for local communities is therefore often cited as one of the reasons for reintroduction where beavers are not yet present (Campbell et al., 2007; Gaywood, 2018; Gurnell et al., 2009; Jones, Halley, Gow, Branscombe, & Aykroyd, 2012; Moran & Lewis, 2014).

There are, however, a number of challenges experienced where beaver and humans interact. In Europe, these are observed mostly where beaver impacts interact with human interests within the riparian zone (Campbell‐Palmer et al., 2016; Halley et al., 2012; Heidecke & Klenner‐Fringes, 1992), particularly in upper and marginal reaches of watercourses where beaver will undertake the largest‐scale habitat alteration (Graham et al., 2020; Halley et al., 2012). For example, where water is stored behind beaver dams, it may inundate land owned by humans which could lead to a financial cost, especially when associated with agriculture or forestry (Campbell‐Palmer et al., 2016; Gaywood et al., 2015; Morzillo & Needham, 2015; Parker et al., 1999). Other notable impacts can include beaver burrow collapse and bank erosion in agricultural land (Campbell‐Palmer et al., 2016; Gurnell, 1998), beaver grazing on arable crops (Campbell‐Palmer et al., 2016, p.; McKinstry & Anderson, 1999), or the felling of particular trees of human importance (Campbell‐Palmer et al., 2016; Campbell‐Palmer, Schwab, & Girling, 2015). Perhaps not surprisingly, beaver are perceived more negatively by people where these conflicts occur (Enck et al., 1992; Jonker et al., 2010; McKinstry & Anderson, 1999; Payne & Peterson, 1986).

Practical management interventions exist that can be employed to address these factors, including dam removal, bank stability management, flow device installation (to lower water levels), tree protection, restoration of riparian zone as management, supported further by compensation or positive incentive payments (Campbell‐Palmer et al., 2015; Campbell‐Palmer et al., 2016; Morzillo & Needham, 2015; Pollock et al., 2017). To reduce the potential for further conflicts, however, particularly those that occur between people over species management (Marshall, White, & Fischer, 2007; Redpath, Bhatia, & Young, 2015), it is recognized that engaging with affected individuals and sharing in the decision‐making processes for management of beaver is vital (Coz & Young, 2020; Decker et al., 2015, 2016; Redpath et al., 2015).

A recent study of local peoples' attitudes toward beaver in Romania and Hungary demonstrated that beaver was often viewed negatively when related to provisioning ecosystem services but positively regarding regulatory or cultural services. As such the study called for recognition of this complexity in perceptions to minimize conflicts, through “reciprocal learning” between conservationists and locals in adaptive management (Ulicsni, Babai, Juhász, Molnár, & Biró, 2020). For beaver, there are a number of management frameworks which seek to engage with affected parties across Europe in a variety of ways, for example: in Bavaria (Germany), regional authorities employ two beaver managers to oversee a network of volunteer beaver consultants throughout the region (Pillai & Heptinstall, 2013; Schwab & Schmidbauer, 2003); in the Netherlands, the government monitors the beaver population and provides management advice to landowners (Pillai & Heptinstall, 2013); in France, the state authorities provide an advisory service at a catchment scale (Campbell‐Palmer et al., 2015; Campbell‐Palmer et al., 2016; River Otter Beaver Trial, 2019). However, although engagement is a key component of management strategies, there are to date, few European studies describing attitudes towards beaver (Ulicsni et al., 2020).

The case is different in Great Britain where beaver is currently being reintroduced at a politically devolved level (with the reintroduction status at varying stages throughout the nations) as there have been a number of studies of attitudes towards the species. This may be because an understanding of social factors is a requirement of reintroduction according to the guidelines set by the International Union for the Conservation of Nature (IUCN & SSC, 2013); these guidelines were published in 2013 after many of the reintroduction projects in mainland Europe (Halley et al., 2012), and of course, these guidelines do not apply to established or naturally dispersing populations of beaver that were not therefore “reintroduced”. Additionally, there is a recent increase in recognition in the literature that the human dimension of environmental projects is a key component of their success or failure (Bennett et al., 2017a, 2017b; Chan et al., 2007; IUCN & SSC, 2013; Redpath et al., 2015). For example, conflicts between humans and wildlife, or between humans about wildlife, may result in threats to species populations or the future success of any attempted species reintroduction (Dickman, 2017; Manfredo & Dayer, 2004; O'Rourke, 2014).

The British studies of attitudes may have limitations (most notably the ability to which they can be deemed representative of a wider population), but they have consistently demonstrated a majority in favor of beaver projects, ranging between 63 and 95.19% of respondents (Auster, Puttock, & Brazier, 2019). However, the intricacies of the social debate run deeper than a simple “for or against” question. A nationwide survey found an association between support for reintroduction and a positive view of potential impacts, and vice versa (Auster et al., 2019). The respondents from the occupational sectors of “Farming and Agriculture” or “Fisheries and Aquaculture” were less likely to have a favorable view of beaver impacts and were thus often (though not unanimously) opposed to beaver reintroduction, which is in line both with other studies conducted in Great Britain (Auster, Barr, & Brazier, 2020a; Crowley, Hinchcliffe, & McDonald, 2017; Gaywood, 2018; Lang, 2004; Scott Porter Research and Marketing Ltd, 1998) and the aforementioned conflict challenges which have been observed across mainland Europe.

Socially, when whomever gains or losses from beaver reintroduction is examined it is concluded that (in certain scenarios) those people who experience the benefits may differ from those who experience the costs (Brazier et al., 2020; Gaywood, 2018). Although it is often cited that the potential benefits of beavers will outweigh the costs (Brazier et al., 2020; Campbell et al., 2007; Gaywood, 2018; Gaywood et al., 2015; Gurnell et al., 2009; Jones et al., 2012; Tayside Beaver Study Group, 2015), the costs that do occur may be attributed to a small number of people who themselves derive little or no direct financial benefit. This distinction between potential beneficiaries and the negatively impacted parties is perhaps most easily demonstrated in the case of beaver damming, where a downstream community may benefit significantly from flood alleviation while the landowner upstream may experience flooding on their property. Thus, strategic management decisions will need to consider how to bridge this disconnect and address potential conflict issues while allowing for the potential opportunities for biodiversity, flow attenuation, water quality, and ecotourism to be maximized.

It is highlighted herein, that to enable maximization of the opportunities from beaver reintroduction that are reviewed above, these conflicts will need to be appropriately recognized; the best management strategies are those where issues are mutually addressed between wildlife management authorities and stakeholders (Auster, Barr, & Brazier, 2020b; Redpath et al., 2015; Rust, 2017; Treves, Wallace, & White, 2009). There are real opportunities resulting from beavers, as discussed above, but there are real conflict challenges to be addressed as well, and they should be considered as one within a holistic approach with a closed‐loop between the beneficiaries and the negatively affected. Further, in the case of reintroduced beavers, such management considerations will need early attention if the potential for later conflicts is to be reduced, particularly as challenges may not yet exist but could occur post‐introduction (Auster et al., 2019; Conover & Decker, 1991; Coz & Young, 2020).

Finally, holistic management strategies will need to incorporate effective communication to aid the reduction of potential conflict issues. In a case from Poland, beavers had been reported as of concern by fishery managers, who cited damage to pond levees. Some of the participants had received compensation for reported damage, but a number of fishery managers had undertaken both authorized and unauthorized beaver culls as the beavers were viewed as problematic. In this scenario, it was reported that “poor communication” by conservation bodies was a particular part of the problem, with a lack of information on management measures and unresponsiveness from government agencies being factors which were suggested to have exacerbated conflict (Kloskowski, 2011). However, the literature recognizes that, when stakeholders are appropriately engaged and communication is effective, trust can be fostered between stakeholders and the wildlife management authorities (Decker et al., 2015, 2016; Redpath et al., 2015; Rust, 2017; Treves et al., 2009). This in turn can enable an environment within which, as Redpath et al. remarked in 2013, wildlife management issues and decisions can be “shared as one” (Redpath et al., 2015).

#### 3.2.1 Summary of human–beaver interactions

- There are real opportunities for humans provided by beavers, as well as real potential conflicts between humans and the activity of beavers. The opportunities may be realized by different people to those who incur the costs in certain contexts.
- Effective management strategies should consider the beneficiaries and cost‐bearers in a holistic manner, bridging the distinctions within a closed‐loop management system.
- Management strategies require clear communication to gain trust between stakeholders and the wildlife management authority, thus providing an environment that is conducive toward addressing issues as a collective and reducing the potential for conflict between parties.

#### 3.2.2 Human–beaver gaps in understanding

- Where they are reintroduced, living with beavers (and associated management) will be a new concept. How do people learn and adapt to this change?
- In policy, what is the best approach for a closed‐loop management framework that maximizes opportunities, for example, ecosystem service provision, while minimizing the potential for conflicts?
- What is the best way to disseminate information regarding approaches to management?

## 4 CONCLUSION: FUTURE SCENARIOS AND CONSIDERATIONS

The beaver is clearly the very definition of a keystone species. The myriad ways in which it alters ecosystems to suit its own needs, which in turn supports other species around it, demonstrate its value in re‐naturalizing the heavily degraded environments that we inhabit and have created. The impacts of beaver reintroduction reviewed herein; to deliver changes to ecosystem structure and geomorphology, hydrology and water resources, water quality, freshwater ecology and humans, and society are profound. Beaver impacts are not always positive, at least from a human perspective, thus it remains critical that the knowledge gaps identified above are addressed as beaver populations grow, to ensure that improved understanding coupled with clear communication of beaver management can prevail.

Where beavers do deliver positive change, on balance benefits are shown to outweigh the costs associated with beaver reintroduction or management. It is unlikely that any other species, including humans, will deliver these changes, thus it would seem rational to conclude that beaver population expansion should be supported, wherever habitat is suitable and the species naturally occurred historically. Indeed, it is suggested that reintroducing beavers, is a genuine example of “working with natural processes” or implementing “nature‐based solutions”, which are both low cost and multi‐faceted. As such, beaver reintroduction can underpin approaches to reverse the decline of species extinctions while also delivering ecosystem services, which may increase resilience to climate change and mitigate associated risks such as flooding and drought.

Of course, such an environmentally progressive approach needs to be implemented hand‐in‐hand with an appropriate management regime, ideally funded by Government, to capitalize on the environmental goods and services that beavers provide, and established as part of a national (or even international) strategy for the reintroduction of the beaver. Such management approaches have been normalized in places such as the German state of Bavaria, where beavers now deliver the wide range of ecosystem services reviewed above, with a pragmatic and flexible approach towards beaver management to support people who experience negative impacts while supporting a favorable conservation status of the species (Pillai & Heptinstall, 2013; Schwab & Schmidbauer, 2003). Other countries, including GB where beaver populations are in their infancy, but expanding, would do well to adopt similar management strategies (e.g., see the River Otter Beaver Trial, 2019) to ensure that successful reintroduction of beavers maximizes the environmental opportunities and minimizes the social conflicts that may manifest (Box 1).

**Case study: Hydrology and water quality—Devon Beaver project**

**FIGURE 4:**
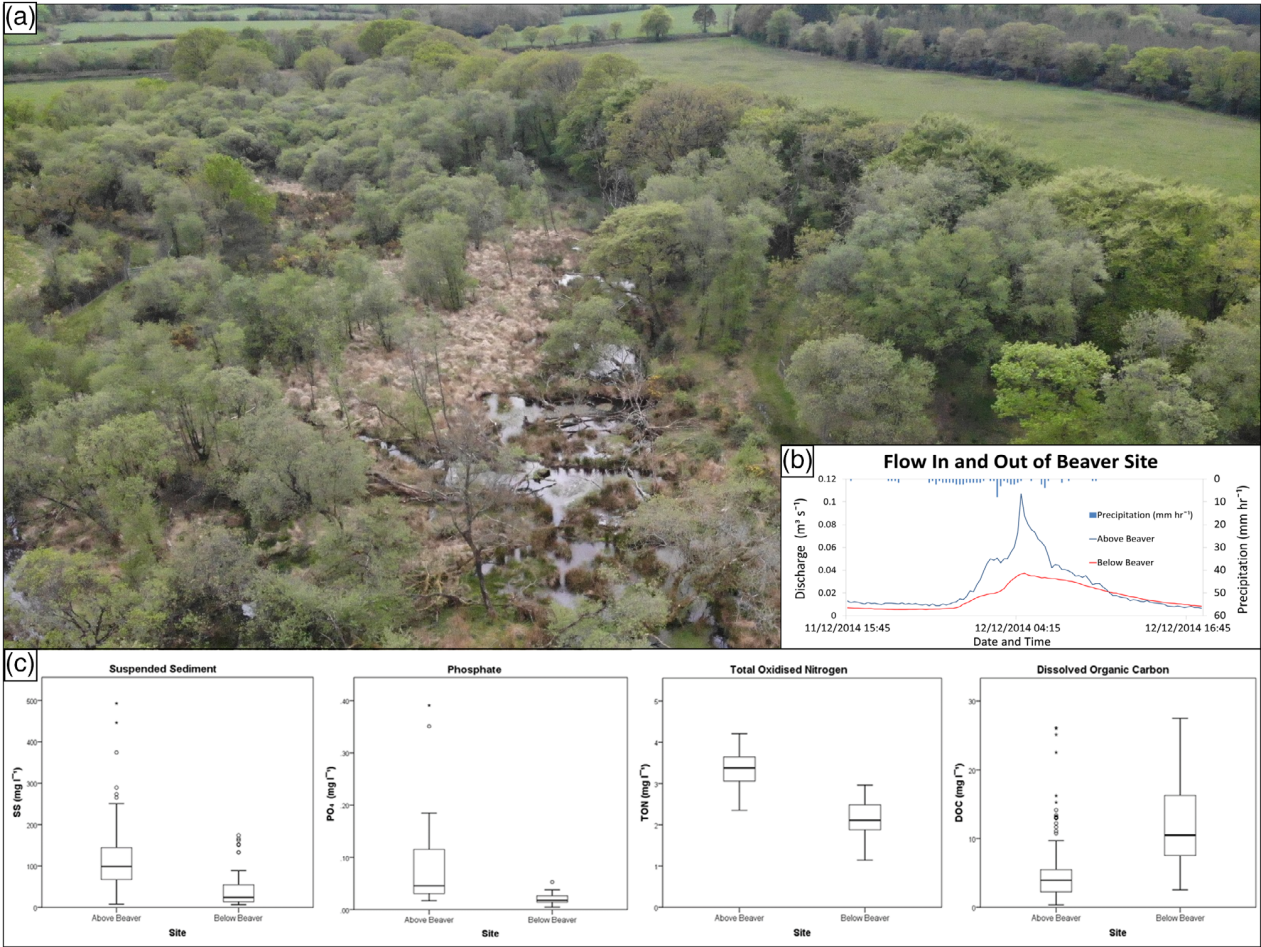
A summary figure for the Devon Beaver Project: (a) aerial photo showing the beaver wetland nestled amongst an agriculturally dominated landscape; (b) an example hydrograph showing the contrast in flow regime between water entering the site (blue) and water leaving the site (red); (b) summary water quality results from the site for each figure “Above Beaver” to the left is the concentration entering the site and “Below Beaver” to the right is concentration leaving the site. From left to right: suspended sediment, phosphate, total oxidized nitrogen, and dissolved organic carbon

Puttock et al. (2017) undertook research at an enclosed and therefore controlled beaver reintroduction site in Devon, South West England. The site is situated on a first‐order stream. In March 2011, a pair of Eurasian beavers were released into a 3 ha enclosure, dominated by mature willow and birch woodland, in addition to gorse scrub. Upstream, the site was fed by a 20 ha catchment area dominated by intensively‐managed grassland. As illustrated in Figure 4, beaver activity at the site created a complex wetland, dominated by 13 ponds, dams, and canal networks (Puttock, Cunliffe, Anderson, & Brazier, 2015). Flow was monitored upstream and downstream of the beaver ponds.

Monitoring of the site between 2013 and 2016 showed that the 13 ponds covered >1,800 m^2^ and stored >1 million liters of water. Across 59 rainfall‐runoff storm events, the outflow below the beaver impacted site showed a more attenuated response relative to water entering the site. Events exhibited on average 34% lower total event discharges, 30% lower peak discharges, and 29% longer lag times below the beaver dam sequence, in contrast, to flow entering the site. Critically, Puttock et al. (2017) analyzed a sub‐set of the largest flood events of greatest interest from a flood risk management perspective. Results showed the flow attenuation impact to persist. Additionally, while the inflow to the site was ephemeral, drying up during drought periods, the outflow from the site never dried up during the monitoring period, highlighting the ability of increased water storage in beaver wetland environments to maintain base flow in river systems.

Analysis was undertaken into sediment storage within the site and water quality entering and leaving the site. A site survey (Puttock et al., 2018) showed that ponds held over 100 t of sediment, 15 t of carbon, and 1 t of nitrogen. Pond size was shown to be the greatest control over storage, with larger ponds holding more sediment per unit area. Source estimates indicated that >70% of the sediment trapped in the ponds was from the upstream agriculturally dominated catchment. A summary of water quality results taken during rainfall‐runoff events (see Puttock et al., 2017) showed that on average, compared to water entering the site, water downstream of the beaver dam sequence contained 3 times less sediment, 0.7 times less nitrogen, 5 times less phosphate, but twice the dissolved organic carbon content. Associated flow attenuation was shown to result in further reductions in total loads.

## CONFLICT OF INTEREST

The authors have declared no conflicts of interest for this article.

## AUTHOR CONTRIBUTIONS

**Richard Brazier:** Writing‐original draft; writing‐review and editing. **Alan Puttock:** Writing‐original draft; writing‐review and editing. **Hugh Graham:** Writing‐original draft; writing‐review and editing. **Roger Auster:** Writing‐original draft; writing‐review and editing. **Kye Davies:** Writing‐original draft; writing‐review and editing. **Chryssa Brown:** Writing‐original draft; writing‐review and editing.

## RELATED WIREs ARTICLES

Of wood and rivers: Bridging the perception gap

Ecosystem engineers in rivers: An introduction to how and where organisms create positive biogeomorphic feedbacks

Reintegrating the North American beaver (Castor canadensis) in the urban landscape

Catchment systems engineering: An holistic approach to catchment management
